# Natural Rubber Nanocomposites: A Review

**DOI:** 10.3390/nano9010012

**Published:** 2018-12-22

**Authors:** Liliane Bokobza

**Affiliations:** 196 Boulevard Bineau, 92200 Neuilly-Sur-Seine, France; Liliane.Bokobza@wanadoo.fr; Tel.: +33-1-4637-2427

**Keywords:** nanomaterials, in situ generated particles, clay particles, carbon nanomaterials, natural rubber, reinforcement

## Abstract

This paper reviews studies carried out on natural rubber filled with nanofillers such as spherical silica particles (generated by the sol gel reaction), clays and carbon nanostructures. It is shown that the mechanical response of NR is influenced by several parameters including the processing conditions, the state of filler dispersion, the polymer-filler interactions and the filler morphological aspects. Even if the sol gel process conducted in vulcanized rubber yields almost ideal dispersions, rod-shaped particles such as clay, carbon fibers or carbon nanotubes are by far more efficient in terms of mechanical reinforcement on account of their anisotropic character and their ability to orientate in the direction of stretch. The efficiency of layered fillers such as clays or graphitic structures clearly depends on the way they are dispersed (exfoliated) in the rubber. Complete exfoliation still remains difficult to achieve which limits the tremendous nanoreinforcement expected from a single layer of clay or graphite. In all cases, the onset of crystallization is observed at a lower strain value than that of the unfilled matrix due to strain amplification effects.

## 1. Introduction

Natural rubber (NR) known as cis-1,4-poly(isoprene) is contained primarily in the milky sap or latex of the *Hevea brasiliensis* tree in addition to a small amount of nonrubber products including proteins, carbohydrates, lipids and inorganic salts. It is isolated by acid coagulation, washed with water then processed into sheets. Improvement in the rubber elasticity and strength is obtained by the vulcanization process usually in the presence of sulfur, accelerator and other compounding ingredients, resulting in a three-dimensional network.

Due to the strain-induced crystallization phenomenon, vulcanized natural rubber (NR) displays high values of tensile strength and elongation at break. This phenomenon that can be considered as a "self-reinforcing effect" is usually attributed of its uniform microstructure (cis-configuration of the macromolecular chains). The presence of crystallites acting as additional cross-links in the network structure and their alignment in the direction of stretch, explain the outstanding mechanical properties of NR [1,2].

In most applications, NR is compounded with fillers, to improve the mechanical response as well as the electrical and thermal conductivities and the barrier properties. The importance of carbon black fillers (CB) in the rubber industry has been known for many years. Reinforcing carbon blacks consist of primary filler particles fused together to build up stable aggregates which stick to form agglomerates that can be destroyed by mechanical mixing. 

The basic processes contributing to the reinforcement effect of a filled rubber have been widely discussed in the literature [3,4,5,6,7]. They include an increase in stiffness attributed to the inclusion of rigid filler particles, an increase in the cross-linking density by polymer-filler interactions and filler-filler interactions that lead, at high filler concentrations, to the formation of a continuous filler network held by weak van der Waals forces. 

Filled rubbers display two hysteretic mechanisms related to energy dissipated by the material during deformation. The first one concerns the non-linear viscoelastic behavior known as the Payne effect because it was extensively studied by Payne [8,9,10]. This effect, characterized by a drop in the storage modulus under dynamic strain conditions, has been mostly explained by a breakdown of the filler network or filler agglomerated structures originating from filler-filler interactions. The electrical conductivity of the composite has also been shown to be affected in a similar way to the modulus changes when the sample is subjected to an increasing dynamic strain amplitude thus showing that electrical conductivity and dynamic shear modulus are dependent on the filler network structure [10]. The Payne effect is obviously linked to agglomeration of the filler particles and the reduction of filler agglomeration reduces the amount of enegy dissipated during deformation thus providing a lower rolling resistance of automative tires. The reduction of rolling resistance for energy saving purposes is a major criterion when developing a rubber compound. Silica has a low affinity with hydrocarbon-based polymers but it can be used in combination with a coupling agent that establishes molecular bridges at the interface between the polymer matrix and the filler surface. In that case, it reduces the amplitude of the Payne effect and consequently the rolling resistance as a result of a better filler dispersion [11,12]. The second hysteretic mechanism is the "stress-softening effect" or Mullins effect [13,14,15] observed at high extensions and characterized by a decrease in stress when the filled sample is extended a second time. Despite the fact that there is no general agreement on the origin of this phenomenon since it can arise from different mechanisms, it has been shown that, in composites filled with conventional fillers (carbon black or silica), the amount of softening depends on the strength of the interactions between the polymer and the filler particles [16]. In the case of a strong interface with highly reactive components, the Mullins effect, associated with the rupture properties of the material, is attributed to a loss of elastic chains that have reached the limit of extensibility by strain amplification effects caused by the inclusion of undeformable filler particles [17]. By tracking the filler structures under uniaxial strain, it was shown that overstraining effects depend on the local filler concentration [18]. In filler-rich areas, the rubber matrix is highly overstrained and chains that connect filler particles reach their limit of extensibility at low strains and detach from the filler surface causing the loss of elastic chains. The state of filler dispersion that determines the amplitude of chain overstraining is of crucial importance for the mechanical response of the polymer composites.

Nanofillers have concentrated huge attention as reinforcing agents for elastomeric materials because they are expected, if well dispersed in the host medium, to offer a large interfacial area with the polymeric medium, available for polymer-filler interactions. Spherical particles as those generated by the sol-gel process, layered fillers such as clays or graphitic structures, single layer of graphite such as graphene, rod-shaped particles such as clay and carbon fibers or carbon nanotubes, are playing an increasingly important role in the development of rubber nanocomposites. Layered and rod-shaped particles with high aspect ratio can form an interconnecting filler network at a much lower filler loading than conventional fillers. This filler network has been shown to have a strong impact on the mechanical and barrier properties of the composite and on elecrical conductivity in the case of black fillers.

This work presents a brief overview of studies carried out on natural rubber nanocomposites based on fillers of different morphology. It focuses on the manufacturing techniques, the extent of reinforcement provided by each type of particles with a special emphasis on their effect on the strain-induced crystallization process as well as on the prevailing problems of filler dispersion.

## 2. Sol-Gel In Situ Generation of Filler Particles

The usual technique of blending filler particles into the elastomer before the cross-linking reaction often yields agglomerated filler structures and inhomogeneous resulting composites. The sol-gel technique based on the polymerization of an inorganic precursor such as tetraethoxysilane (TEOS) has been shown to yield small and well-dispersed particles within the host matrix. Pioneered by Mark for the generation of silica or titania particles in poly(dimethylsiloxane) (PDMS) [19,20,21,22], it has been extended in other polymers including NR [23,24,25,26,27]. The in situ technique starts by swelling the polymer with TEOS followed by a hydrolysis process in the presence of a catalyst which has a great influence on the morphology of the generated inorganic structures. The sol-gel reaction is the following:

Si(OC_2_H_5_)_4_ + 2H_2_O → SiO_2_ + 4C_2_H_5_OH

The sol-gel process can be carried out before or after the cross-linking reaction. In the unvulcanized state, it can take place in the raw material swollen with the metal oxide precursor or in a solution containing the rubber and all the ingredients of formulation. The filling process carried out in an already preformed network has been shown to yield a very fine filler morphology on account of a steric limitation for cluster growth by the network chains [28,29].

Ikeda’s group [23,24,25,26] conducted the sol gel reaction of TEOS before curing by immersing an unvulcanized NR sheet in TEOS for several hours at room temperature. The swollen sheet was then soaked in an aqueous solution catalyst–typically an amine- and then dried before being subjected to the sulfur vulcanization. The effect of type of amine on the sol-gel reaction of TEOS was investigated and polarity as well as solubility in water were shown to be important parameters in the generated silica content [26]. The stress-strain curve of in situ silica filled NR vulcanizate displays a lower stress at the elongation up to 200% and higher stress at the elongation beyond 200% than a sample filled with a same amount (71 phr, “phr” = parts per 100 parts of rubber by weight) of commercial silica. The high modulus at low strains for the conventional silica filled rubber has been assumed to be due to the formation of large aggregates of silica while the higher tensile strength exhibited by the in situ filled sample is explained by a stronger interaction between the generated particles and the rubber chains. 

Miloskovska et al. [30] have analyzed the chemical structure of silica obtained via the sol-gel reaction using hexylamine as catalyst and TEOS as alkoxy precursor. The in situ generation of silica is conducted in NR and EPDM (ethylene-propylene diene rubber) and the analysis is performed in the uncrosslinked state. Solid-state NMR spectroscopy indicates the presence of hexylamine in both sol-gel synthesized ex situ silica and the in situ synthesized NR nanocomposite. It is assumed that the diffusion limitations (TEOS and hexylamine) inside the rubber matrix leads to a lower degree of particle condensation, to fewer Si-OH groups and residual ethoxy groups. The presence of these ethoxy groups and hexylamine on or near the silica surface in addition to a possible entrapment of rubber chains in the growing particles, contribute to a more hydrophobic nature of the particle surface and a better compatibility with the rubber matrix. 

**Figure 1:**
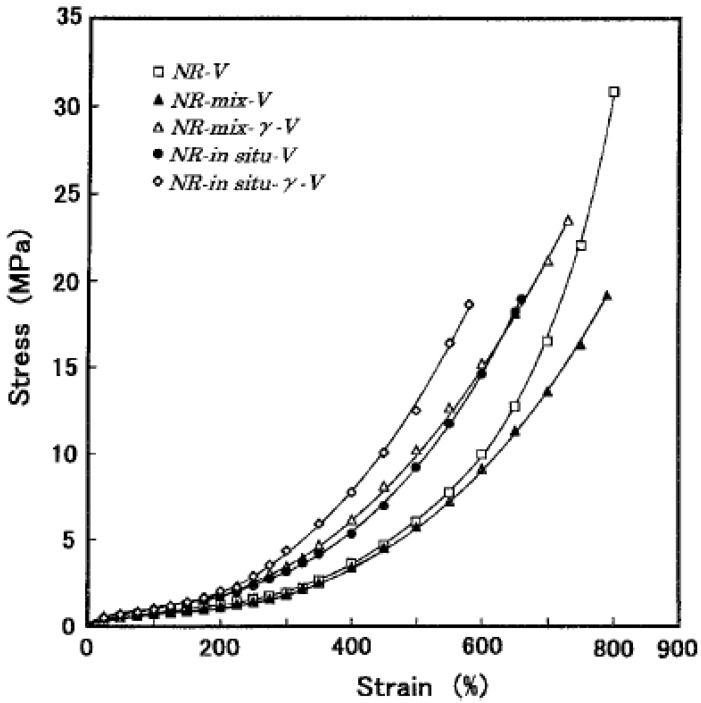
Stress-strain curves of NR vulcanizates at 25 °C: NR-V-unfilled NR; NR-mix-V: NR vulcanizate filled with commercial silica; NR-in situ-V: NR vulcanizate filled with in situ silica; NR-mix-γ-V and NR-in situ-γ-V contain the coupling agent. The amount of filler is 33 phr in filled samples. Reproduced with permission from [31]. Copyright Springer Nature, 2003.

**Figure 2:**
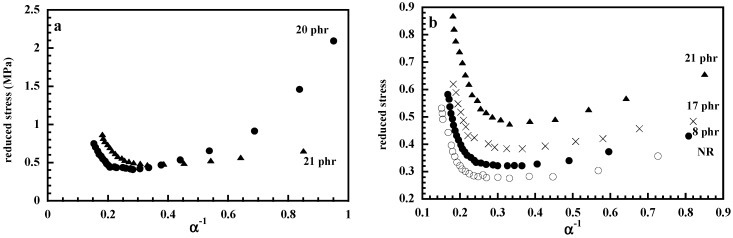
Mooney-Rivlin plots of NR composites: (**a**) submitted to the in situ silica filling process before (•) and after (▲) vulcanization; (**b**) filled in the vulcanized state and in comparison with the unfilled NR. Reproduced with permission from [27]. Copyright Elsevier, 2005.

Conventional silica is well known to affect the cross-linking density in vulcanization systems based on accelerator/sulfur cure systems by reacting with the chemical ingredients of formulation [11] and coupling agents are used to overcome this effect. The effect of silane coupling agent (γ–mercaptopropyltrimethoxysilane) on the reinforcement of NR by in situ generated silica that can be carried out only in the unvulcanized state, has been investigated by Murakami et al. [31]. The degree of increase on the stress of in situ silica-filled vulcanizate by adding the coupling agent (NR-in situ-γ-V), is shown to be smaller than that of conventional silica-filled vulcanizate (NR-mix-γ-V), which is ascribed in the difference of numbers of silanol groups on the silica surfaces between in situ and commercial silicas (Figure 1). Bokobza and Chauvin [27] also conducted the sol-gel process but with a synthetic protocol that differs from that of Murakami et al. [31] since the in situ filling of silica is carried out in a toluene solution of natural rubber containing all the ingredients of formulation (zinc oxide, sulfur, stearic acid, cyclohexyl benzothiazole sulfenamide) in addition of TEOS (calculated in order to get a given filler loading), the catalyst (dibutyltin diacetate) and the silane coupling agent (the bis(3-triethoxysilylpropyl) tetrasulfide, commonly abbreviated as "Si69". The use of the coupling agent is seen to provide a substantial reinforcement of the NR matrix but contrary to the results of Murakami et al. [31], a decrease in the cross-linking density with regard to the unfilled formulation is observed in the absence of Si69 due to the reaction of silica with sulfur. The Mooney-Rivlin representation that plots the reduced stress [σ*], defined by the quantity [σ*] = σ / (α − α^−2^) against the reciprocal extension ratio, α, made clearer peculiarities of the stress-strain curve of a filled vulcanizate. The large decrease in the reduced stress observed at low deformation for the sample filled by silica particles in the absence of a coupling agent is attributed to the breakdown of agglomerated filler structures formed by interactions between active silanols present on the silica surface (Figure 2a). 

The in situ filling process has also been conducted in chemically cross-linked NR vulcanizates by simply swelling the moulded film in TEOS in the presence of a tin catalyst (dibutyltin diacetate) at a given swelling time that determines the degree of TEOS absorption and consequently the filler loading [27]. The swollen film is exposed to saturated water vapor then vacuum-dried at 80 °C for removal of remaining metal alkoxide and by-products. This process provides a good dispersion of the inorganic particles evidenced by the absence of the decrease in the reduced stress at low strains (Figure 2b) contrary to the case of composites filled with particles generated in the unvulcanized rubber. On the other hand, particles generated in an already preformed networks impart to the NR matrix a substantial reinforcement despite the fact that it is conducted without the use of a coupling agent. Since NR has no affinity for silica, weak polymer-filler interactions are expected contrary to in situ silica- or titania-filled PDMS composites where PDMS chains interact strongly via hydrogen bonds with the silanols present on the particle surface [28,29]. In these PDMS nanocomposites, the polymer-filler interactions lead to immobilization of chain segments resulting in a rubber shell of a few nanometers thick in which chain mobility is reduced with regard to that of the polymer matrix [28,32,33,34]. Differential scanning calorimetry and dielectric techniques have shown that even in the absence of specific interactions between NR chains and silica, polymer segments near the filler surface exhibit restricted segmental mobility characterized by slower segmental relaxation times by 2–3 order of magnitude with regard to bulk behavior [35]. This slowing down of the dynamics, less pronounced than in PDMS/silica nanocomposites, can be understood as a type of confinement of polymer chains on the particle surface. The interfacial dynamics appear unaffected by the silica phase in aggregated NR composites as those prepared by conducting the sol-gel process before the cross-linking reaction [35]. A recent solid state NMR study of polyisoprene samples filled with in situ generated silica indicates the presence of loosely bound rubber only experiencing a larger degree of mobility than that of a tightly bound rubber [36].

An interesting feature of the Mooney-Rivlin plots displayed in Figure 2b for unfilled NR and for composites filled in the vulcanized state, is the strong increase in stress at high deformations, attributed to the strain-induced crystallization of polymer chains. One can see that the crystallization process starts at a lower deformation in the presence of silica which may most probably arise from strain amplification effects due to the inclusion of undeformable filler particles that cause overstraining of polymer chains [37]. 

Tangpasuthadol et al. [38] report the synthesis of in situ silica-NR composites by mixing TEOS with commercial-graded concentrated NR latex consisting of 60% dry rubber, 40% water and about 0.7% ammonia (by weight). The amount of ammonia, present in the commercial latex, was expected to be sufficient for accelerating the sol-gel process. The TEOS was mixed directly into the latex and after conversion to silica, the mixed latex was left in a 50 °C for drying before sulfur vulcanization. The particle sizes were estimated to be between 100 and 500 nm which seems rather high with regard to the usual size obtained by the sol-gel process and suggests particle aggregation. In a second paper of the same group [39], the sol-gel process of TEOS combined with alkyltriethoxysilanes containing vinyl, ethyl or butyl functional group, was used to generate silica in natural rubber latex. It was shown that the silane conversion to silica in the NR matrix decreased when the alkyl group of the alkyltriethoxysilanes increased in size.

Solvent swelling and viscoelastic responses of epoxidized natural rubber (ENR) with 50 mol % of epoxy content filled with in situ generated silica have been analyzed by Bandyopadhyay et al. [40]. The sol-gel technique was carried out by adding TEOS and HCl (acting as the catalysis) in a solution of ENR in THF and dicumyl peroxide was the curative agent. The authors have investigated the swelling behavior of the filled composites by using the procedure of Kraus [41] consisting of plotting the ratio of the equilibrium swelling ratios in a solvent (Q_r_/Q_r0_) for the filled (Q_r_) and unfilled (Q_r0_) samples against the quantity (ϕ/(1−ϕ)) were ϕ is filler volume fraction. The slope of the curve allows the determination of a parameter, C, that may be taken as a reflect of adhesion between the polymer and the filler. It was shown that the generated silica particles within ENR yields a substantially higher C value than that of the carbon black filled conventional rubber composites. The amplitude of the Payne effect has been shown to decrease with the extent of polymer-filler interactions. In poly(vinyl alcohol)/silica hybrid nanocomposites because of the presence of a large concentration of interactive OH groups over the polymer backbone, the Payne effect is lower than in the ENR/SiO_2_ composites where the OH groups arise from the ring opening reaction of the epoxy group forming a diol structure in the presence of acids. 

Li et al. [42] prepared through the sol-gel method, in situ epoxidized natural rubber/ SiO_2_ hybrids without any curing additives. During a hot pressing process at 60 °C and 145 °C for 20 min, the filled sample was cross-linked via hydrogen bons between Si-OH and C-OH and also between Si-OH and –C=O groups generated by the oxidation of C-OH under hot-press treatment at 145 °C. It is shown that the generated silica imparts to the rubber composites higher modulus and strength than in the case of the traditional mixing method with conventional silica. Moreover, after the hot press treatment in 145 °C, the composite gains a higher modulus and strength as a result of a stronger hydrogen-bond interaction between –C=O/–OH than that of –OH/–OH. 

In conclusion, although the organosilane based sol-gel chemistry, has opened a way for the production, with no health risk, of polymer nanocomposites with nanoscale dispersed particles, no-industrial-scale application of this technique has been developed to date because the rubber, in the vulcanized or unvulcanized states, has to be immersed in large amounts of a precursor of high cost.

## 3. Clay Particles

Layered silicates such as montmorillonite (MMT) which are two-dimendional fillers composed of stacked layers held together by van der Waals forces, have been widely investigated as potential reinforcing nanofillers of elastomeric materials. 

The interest in this type of clays is motivated by a possible obtention of full separation (called delamination or exfoliation) of the nm-thick layers randomly dispersed in a polymer matrix which would provide the most improved mechanical properties at a low filler loading on account of their large specific surface area and, as a consequence, a large interfacial area with the host polymeric matrix.

Before being incorporated in a polymer, the layered silicate has to be modified in order to replace the interlayer cation originally present on the clay surfaces by alkyl ammonium ions. In this way, the silicate layers become organophilic and compatible with the hydrophobic polymer. At the same time, this treatment increases the interlayer distance thus making possible the intercalation of polymer chains into the intergallery spacing. The state of clay dispersion can be evaluated from X-ray diffraction (XRD) measurements of the interlayer distance: intercalation of polymer chains is characterized by an increase in the intergallery spacing while the absence of a diffraction reflects clay exfoliation.

**Figure 3:**
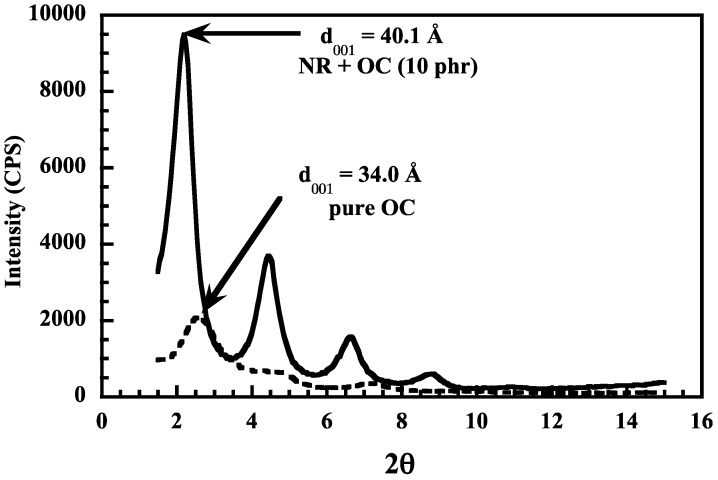
X-ray diffraction patterns for pure organoclay (OC) and for the NR composite filled with 10 phr of OC. Reproduced with permission from [43]. Copyright ACS Publications, 2002.

**Figure 4:**
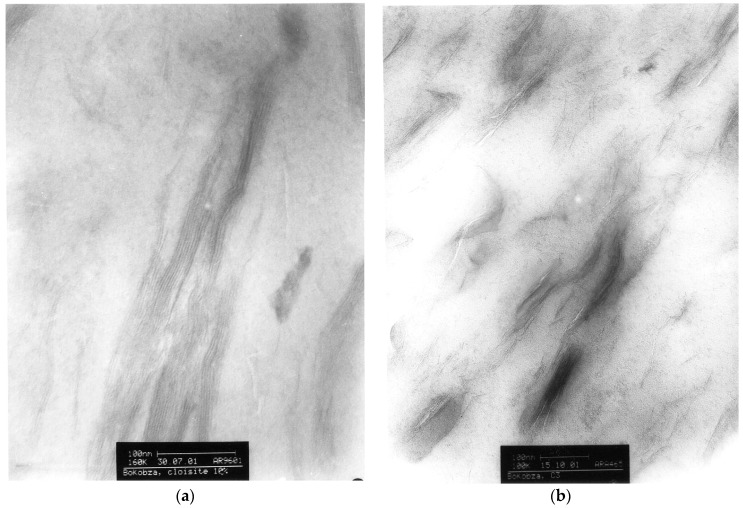
TEM micrographs of natural rubber filled with 10 wt % of clay: (**a**) is for unmodified clay (Reproduced with permission from [43]. Copyright ACS Publications, 2002.) and (**b**) is for organomodified clay. The higher horizontal bar corresponds to 100 nm.

Rubber organoclay nanocomposites can be prepared in solution, in the molten state or by the latex compounding method. Solution blending consists of dissolution of the rubber in a good solvent, addition of clay under stirring then complete evaporation of the solvent followed by the curing process. Joly et al. [43] dissolved NR containing all the ingredients of formulation, in toluene along with 10 phr of an organomodified clay (dimethyl hydrogenated tallow (2-ethylhexyl) ammonium montmorillonite). The X-ray diffraction pattern shows a shift to lower angle values of the diffraction peak of the organoclay dispersed in NR in comparison with the peak of the pure organoclay used, indicating an increase in the interlayer spacing caused by the intercalation of the polymer chains between the silicate layes (Figure 3). Figure 4 shows the TEM micrographs of NR composites filled with 10 phr of the unmodified clay (Figure 4a) and of the organoclay (OC) (Figure 4b). Figure 4a displays typical clay tactoids with layered structures while Figure 4b is that of an intercalated system where the clays retain much of their face-to-face alignments, although a small amount does indeed exfoliate. The organoclay imparts to the rubbery matrix a higher modulus attributed to the high platelet aspect ratio. Birefringence and infrared dichroism measurements under strain show an increase in the orientational level of polymer chains upon incorporation of the clay. On the other hand, the strain-induced crystallization in the unfilled and filled NR has been followed by the shift of the C-H out-of-plane infrared absorption band. 

Varghese and Karger-Kocsis [44] used the latex route to prepare natural rubber-based nanocomposites with pristine layered silicates containing hydrated Na^+^ ion (sodium bentonite and sodium fluorohectorite). These clays are strongly hydrophilic and swell in the presence of water making possible a delamination of the layered structure. The sodium fluorohectorite yields the highest mechanical performance with a high initial stress attributed to the formation of a clay network favored by the large amount of exfoliated layers. Interestingly, the authors mention an anisotropic swelling in toluene due to the orientation of the clay layers. The anisotropic swelling behavior of non-vulcanized NR/NaMMT composites prepared from latex dispersion, was also reported by Valadares et al. [45] who show that upon swelling, the coordinates in the film plane change much less than the coordinate normal to the plane of the film. 

**Figure 5:**
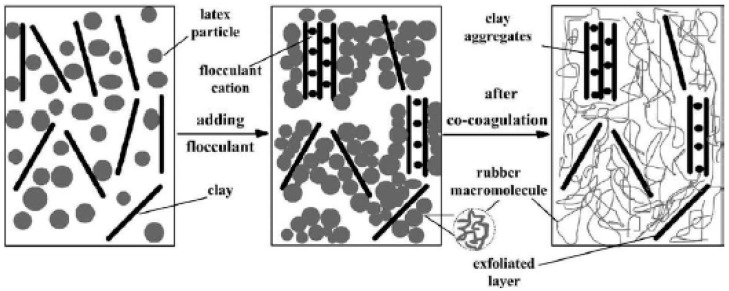
Schematic illustration of the mixing and co-coagulating process. Reproduced with permission from [46]. Copyright Elsevier, 2005.

Wu et al. [46] also used the latex route to prepare rubber-pristine clay nanocomposites by co-coagulating rubber latex and clay aqueous supension. In the aqueous suspension, no X-ray diffraction peak is observed which indicates a complete exfoliation of clay in water. From the XRD measurements of the composites, the authors conclude that the diffraction peaks could originate from the cations of the flocculant in the intergallery and assume that the rubber molecules do not intercalate into the clay galleries. It is thus suggested that during the co-coagulating process, the flocculant coagulate the rubber latex and the silicate layers simultaneously as described in Figure 5.

Rezende et al. [47] also use the "latex method" to prepare non-vulcanized NR reinforced with sodium MMT. Mechanical and structural properties are examined in non-dialized NR and also in the rubber that has been dialized in order to remove excess ionic species and small non-rubber components present in the latex particles. Transmission electron microscopy (TEM) and small angle neutron scattering (SANS) reveal co-existence of individual clay platelets with tactoids in the dialized samples and a lower degree of exfoliation in the non-dialized rubber explained by the high ionic strength and the presence of multivalent cations that promote attractions of clay lamellae. It is shown that the mechanical reinforcement effect is much higher in dialyzed rubber related to an effective aspect ratio f ~200–300 within the expected range of values for single clay platelets.

Melt blending by simply compounding rubber with clays is by far the most common technique for the synthesis of rubber clay nanocomposites both in industry and in laboratory. Galimberti et al. [48] showed that blending a layered pristine clay (Na^+^-MMT) with an ammonium salt organic modifier (di(hydrogenated tallow)-dimethylammonium chloride) in the presence of NR, leads to clay intercalates with organic bilayers with an interlayer distance of 6.0 nm. This high distance, much higher compared to 1.2 nm for pristine clay, may explain the formation of the exfoliated clay by thermal decomposition at 275 °C of the organic modifier.

Carretero-Gonzales et al. [49] carried out in situ stress-strain experiments coupled with synchrotron X-ray diffraction to investigate the effects of an organoclay on strain-induced crystallization of NR composites under uniaxial stretching. It is shown that the nanoclays became highly oriented along the direction of strain and that this orientation starts at a relatively low strain. On the other hand, significant enhanced crystallinity and a lower strain value for the onset of crystallization are reported. Moreover, the authors mention two well-defined crystallization steps for the NR composites contrary to the unfilled sample that exhibits a single crystallization step. The crystallization of the unfilled NR in most studies appears at an extension ratio around 4 and that of vulcanizates filled with 45 or 50 phr of carbon black is found around 2 [50,51]. The crystallization onset in filled vulcanizates is attributed to the strain amplification phenomenon according to which the effective extension ratio of the rubber part is larger than the macroscopic one. Poompradub et al. [52] consider that taking into account the amplification factor for the filled sample leads to a crystallization onset strain similar to that of the unfilled sample. The amplificator factor is usually considered to be the same in the whole portion of rubber but as mentioned previously, it is highly dependent on the local filler concentration that determines the local stress field around polymer chains [18]. In the work of Carretero-Gonzales et al. [49], 15 phr of organoclay (OC) in NR yields an onset of crystallization strain of deformation-induced crystallization of 1.2 which is much lower than that observed in conventionally NR composites. Besides the overstraining of polymer chains by the strain amplification effect, the strong orientation of the nanoclays along the stretching direction also favors the orientation of polymer chains thus promoting nucleation under stretching. 

**Figure 6:**
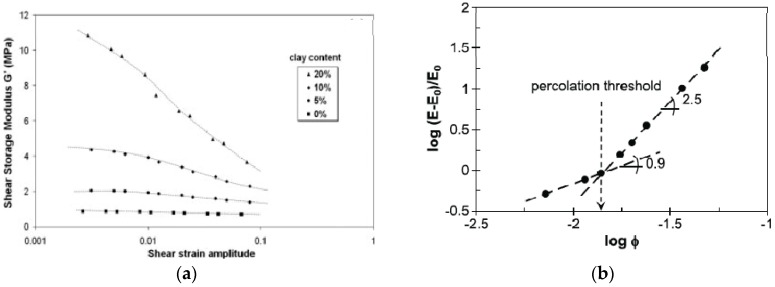
(**a**): Dynamic storage modulus plotted against strain amplitude for NR/OC samples, Reproduced with permission from [53]. Copyright Wiley, 2007; (**b**): Double logarithmic plot of the excess modulus (E − E_0_) with respect to the modulus of the unfilled vulcanizate E_0_ against the filler volume fraction, Reproduced with permission from [54]. Copyright Elsevier, 2009.

In an analysis of the dynamic and viscoelastic behavior of NR/organomodified MMT nanocomposites obtained by melt blending, Ramorino et al. [53] report a nonlinear behavior of the storage modulus with shear strain amplitude (Payne effect) that increases strongly with the clay content (Figure 6a) and is considerably more pronounced than in natural rubber filled with similar loadings of conventional fillers. Static mechanical measurements also reveal that the dependence of the initial modulus on the clay content is strongly nonlinear and suggests two different regimes, under and above the filler percolation threshold [54]. In CB-filled rubber where the aggregates form fractal structures, the excess modulus (E − E_0_)/E_0_ (where E and E_0_ are the initial modulus of filled and unfilled rubber, respectively), has been found to scale linear with the filler volume fraction under the percolation threshold, ϕ_P_, and according to a power law with exponent 4 above ϕ_P_ [55]. Applying the same representation to the NR/OC systems led to exponents equal to 0.9 and 2.5 in low and high filler contents respectively [54] which is quite similar to the values obtained in SBR/OC samples [56]. The intersection of the two straight lines identifies the percolation threshold at ϕ_P_ equal to 0.014 corresponding to 8 phr (Figure 6b).

**Figure 7:**
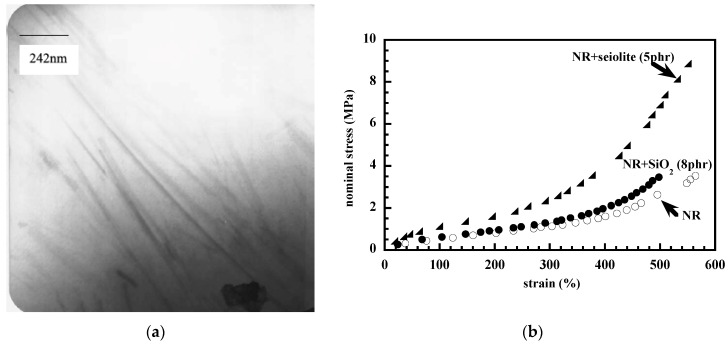
NR/sepiolite (5phr) composite: (**a**) TEM micrograph (Reproduced with permission from [27]. Copyright Elsevier, 2005); (**b**) stress-strain curve in addition to that of the unfilled rubber and with NR filled with 8 phr of in situ precipitated silica.

**Figure 8:**
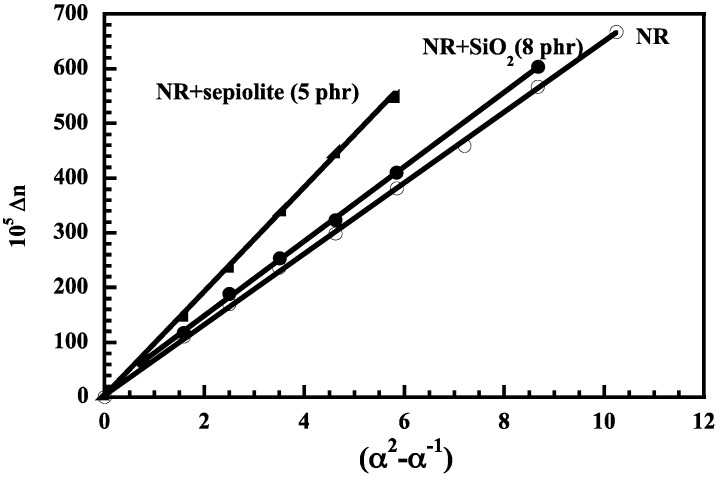
Birefringence measurements for unfilled NR and for composites. Reproduced with permission from [27]. Copyright Elsevier, 2005.

Sepiolite has also attracted interest (although much less than montmorillonite) as reinforcing filler for NR on account of its fiber-like structural morphology [27,57,58]. It is a hydrous magnesium silicate, with a crystal structure formed by two sheets of tetrahedral silica units bonded to a central sheet of magnesium atoms. Internal channels run along the entire length of the structure. The microfibres of sepiolite stick together and form bundles but a micronization process can be applied to disagglomerate the microfibers. Like montmorillonite, the sepiolite surface can be modified in order to make it more compatible with polymers. NR composites containing an organophilic seiolite (Pangel B20 from Tolsa) were prepared by Bokobza and Chauvin [27] by solution blending. They are seen to display a good dispersion where the particles are not aggregated and exhibit their needle-like shape (Figure 7a). Stress-strain measurements in Figure 7b, show that at a similar filler loading, nanofibers of sepiolite impart to the rubbery matrix a much higher reinforcement than the spherical silica particles as a result of the high anisotropy and high orienting capability of the acicular fillers. The orientation of the sepiolite particles in the direction of strain induces an orientation of the polymer chain in the direction of the fiber. Chain orientation under strain can be evaluated by infrared dichroism or biregringence which have been widely discussed elsewhere [59,60]. Birefringence measurements displayed in Figure 8 reveal a higher level of NR orientation for the composite filled with sepiolite. Very briefly, in its simplest form, the birefringence Δn is expressed as the product of two contributions: a front factor inversely proportional to the number of bonds in the chain between physical and chemical junctions and the strain function (α^2^−α^−1^) where α is the extension ratio. Interestingly, it is also demonstrated that the high aspect ratio of the sepiolite particles gives rise to an anisotropic swelling of the rubber phase since the values of the equilibrium swelling ratio in toluene are 2.91 and 4.13 when evaluated from the length or the thickness of the sample, respectively [27]. On the other hand, the high decrease of the swelling ratio with regard to that of the unfilled rubber (equal to 5.3) and silica-filled NR, reflects adhesion of polymer chains along the clay fiber.

In a paper intended to demonstrate that clay particles could be organically modified in situ during mixing rather than using a pre-modified organoclay, Lowe et al. [58] have prepared NR/clay nanocomposites by melt compounding NR with unmodified montmorillonite or sepiolite in addition to the distearyldimethylammonium chloride (DDAC) used as the modifying agent. For both clays, the microstructure, as revealed by TEM, is almost identical to that of nanocomposites made using pre-modified organoclays. It appeas that clay modification in situ has the same efficiency than pre-modified organoclays which makes easier the production of the composites only based on pristine clays. The authors show that, contrary to montmorillonite, unmodified sepiolite (Pangel S9 from Tolsa) disperses well in NR but the tensile modulus of sepiolite-filled NR is increased by addition of DDAC and is similar to that of the composite filled with the pre-modified organoclay (Pangel B20 from Tolsa) thus suggesting that in situ modification is comparable to pre-modification for sepiolite. 

Bhattacharya et al. [57] have discussed the effect of different nanofillers (montmorillonite, hectorite, laponite, sepiolite, silica, carbon black, expanded graphite and carbon nanofibers) on the properties of NR nanocomposites. The effects of filler loading, methods of dispersion as well as chemical modification of the filler particles, have been investigated through the analysis of the mechanical and swelling behavior of the composites, atomic force and transmission electron microscopies, X-ray diffraction and surface energy studies. Modification of an organosepiolite by a titanate acting as a dispersing agent is seen to impart remarkable improvement in the mechanical properties of the NR matrix. It has been ascribed to a synergistic effect of the quaternary ammonium salt of the organosepiolite and the organo content of the titanate coupler. It is also assumed that the silanol groups present on the whole external surface of sepiolite can react with chemical groups of titanate resulting in enhanced compatibilization and efficient interfacial stress transfer.

Hayeemasae and Ismail [61] have incorporated sepiolite into epoxidized NR in an attempt to take advantage of a possible interaction between the hydroxy/siloxane groups of sepiolite and the epoxy groups of the ENR matrix. They report an increase in modulus, tensile and tear strength with sepiolite loading till 5 phr but the improvements are much more weaker than those obtained in NR [27].

## 4. Carbon Nanomaterials

As already mentioned carbon black (CB) is the most widely reinforcing filler in NR formulations. However a large amount of CB is required to reach the desired mechanical and electrical properties of the composite materials. Nowadays, the focus of innovative research is on other forms of carbon including carbon nanotubes, graphite or graphene. These carbon species with fiber, lamellar or single-sheet morphology have attracted considerable attention as fillers for elastomeric compounds on account of their intrinsic mechanical and electrical properties [62,63,64,65,66]. Of particular interest is the high aspect ratio of these conductive fillers that allow, in the case of an optimized dispersion in the host matrix, to reach the percolation threshold at very low filler loadings. On the other hand, their alignment and their interfacial bonding with the polymer chains are also significant parameters in rubber reinforcement. 

**Figure 9:**
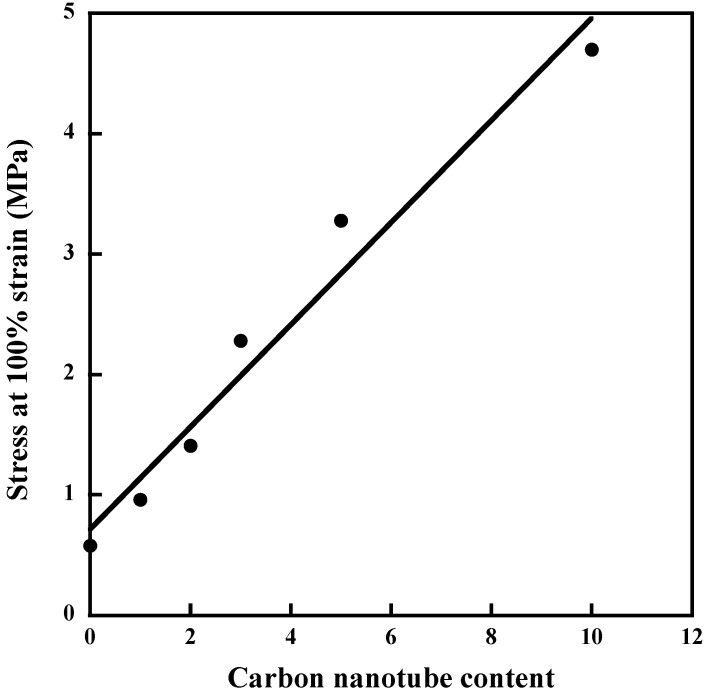
Stress at 100% strain for NR composites filled with multiwall carbon nanotubes (MWCNTs). Reproduced with permission from [69]. Copyright eXPRESS Polymer Letters, 2012.

**Figure 10:**
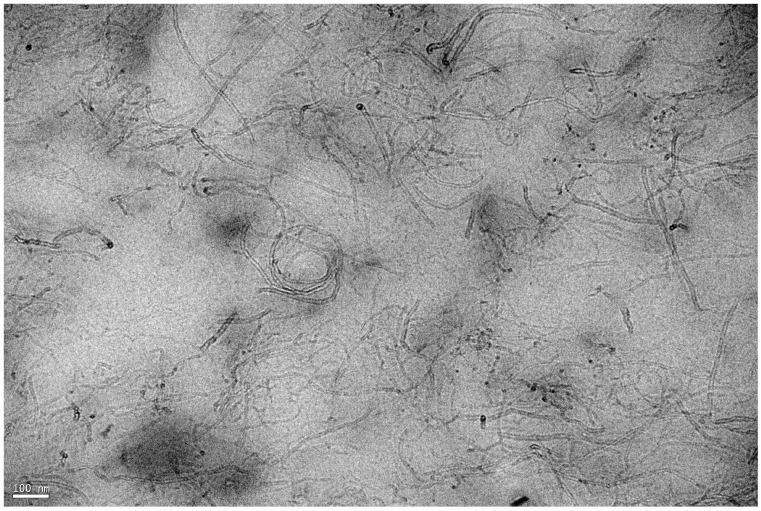
TEM image of NR filled with 3 phr of MWCNTs. The scale bar represents 100 nm.

Of the carbon-based nanofillers, carbon nanotubes (CNTs) have generated numerous investigations on account of their unique properties arising from their one-dimensional character. Substantial Improvements in stiffness have been reported upon addition of multiwall carbon nanotubes (MWCNTs) in NR [67,68,69]. With regard to the pure polymer, 1 phr of MWCNTs incorporated into NR by the sonication method, leads to a 65% increase in the stress at 100% strain. At 10 phr, the increase is more than 700% (Figure 9) and the electrical percolation is reached at less than 1 phr [69]. But the full potential of the tube capability is strongly limited by the presence of bundles (Figure 10) as a result of van der Waals attraction between the tubes. These bundles are responsible of the reduction in the strain at rupture with the nanotube loading. 

Different extents of property improvements and different electrical percolation values are obtained by the use of different processing techniques to produce the nanocomposites [69,70,71]. As explained by Ma et al. [72], dispersion of CNTs in a host matrix is more difficult than the other fillers on account of their high aspect ratio and thus very large surface area. In their paper, techniques including ultrasonication, shear mixing, calendaring, ball mixing, stirring and extrusion, intented to incorporate disentangled CNTs inside a polymer matrix, are presented and discussed. Another approach to get disaggregation and uniform dispersion of CNTs in host matrices is the use of surfactants that adsorb non-convalently on the tube surface [73,74]. This method, contrary to functionalization of CNTs by chemical bonding, does not deteriorate the electronic properties of the tube. The influence of non-covalent functionalization of carbon nanotubes by various surfactants on the rheological behavior of natural rubber latex nanocomposites was investigated by Ponnamma et al. [75]. It was shown that anionic sodium dodecyl sulphate achieves the best dispersion as reflected by the highest Payne effect associated with the formation of a three-dimensional network. It is here of interest to mention that in conventional composites, a good level of filler dispersion is reflected by a decrease in the amplitude of the Payne effect while the contrary is observed with anisoptropic fillers able to form a percolated network at very low filler content if well dispersed in the polymeric medium. As mentioned by Galimberti et al. [76], the large hysteresis or energy dissipation from the Payne effect exhibited by CNTs could be detrimental for dynamic mechanical applications since it contributes to fuel consumption. CNT dispersion in elastomeric matrices has been improved by incorporating another type of filler, namely CB [76,77,78], clays [79] or reduced graphene oxide [80] on account of synergistic effects arising between the two different filler morphologies. The dual filling yields improvement in mechanical and electrical conductivity with a lower percolation threshold than that obtained with composites filled with a single filler [77].

**Figure 11:**
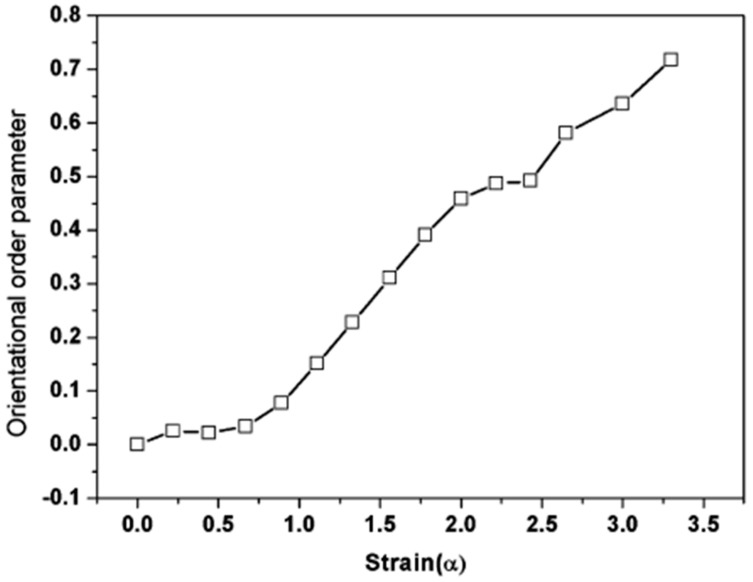
Strain dependence of the orientational order parameter <${\bar{P}}_{2}$> of MWCNTs in the 15 wt % MWCNT-filled NR matrix. Reproduced with permission from [81]. Copyright ACS Publications, 2010.

Regarding strain-induced crystallization of NR, Weng et al. [81] have shown, by using synchroton wide-angle X-ray diffraction, that MWCNTs lead to higher crystallinity and lower onset of crystallization in MWCNT-filled NR than in the unfilled sample. As already observed in nanoclay-filled rubber [49], the results reveals that, contrary to unfilled NR that exhibits a single crystallization step, the MWCNT-composite has a slope similar to that of pure NR in the strain range of 2.8-3.8 and a much larger slope value above 3.8 thus showing that the nanotubes accelerate the crystallization rate at large deformations. The orientational order parameter <${\bar{P}}_{2}$> of MWCNTs in the 15 wt % MWCNT-filled NR has been seen to reach the value of 0.72 at a strain of 3.3 which indicates a remarkable orientational degree of the tubes in the NR matrix (Figure 11). Very briefly, the orientation of structural units of a uniaxially stretched polymer can be described by an orientation distribution that may be written in terms of a Legendre polynomial expansion. The second expansion coefficient <${\bar{P}}_{2}$> or order parameter is equal to 0 in an isotropic structure and to 1 in the case of a perfect orientation along the direction of stretch. The reader will find more details in reference [82]. Mooney-Rivlin plots give also evidence of the shift towards lower strain values of the strain-induced crystallization process in MWNCT-filled NR composites attributed to local strain amplification phenomenon [66,69,83].

Graphene is a single layer of sp^2^ bonded carbon atoms packed into a two-dimensional honeycomb lattice. In recent years, it attracted an enormous interest as a nanofiller on account of its outstanding properties such as the exceptional modulus and the remarkably high electrical and thermal conductivities. These intrinsic properties, associated with the single-layer morphology and better than those of other carbon nanomaterials, have launched a new dimension for the emergence of ultra-lightweight, low cost, and advanced composite materials [84,85]. But like other nanomaterials, the state of graphene dispersion is of crucial importance for the physical performance of the elastomeric material. The key challenge to take advantage of the unique properties of the individual graphene sheets is to avoid, during the synthesis of graphene-filled composites, restack of the single layers through van der Waals interactions.

**Figure 12:**
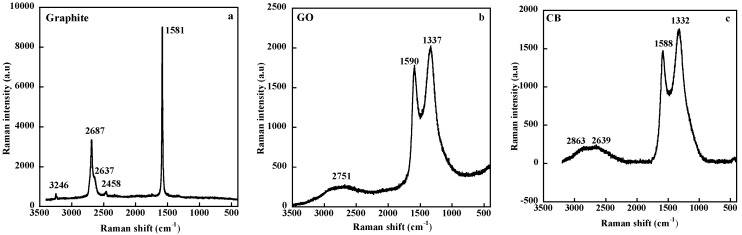
Raman spectra of graphite (**a**), graphene oxide (**b**) and carbon black (**c**) excited at 633 nm.

Different approaches have been used to obtain graphene nanosheets but the graphite oxide route, well described by Kuilla et al. [84] and consisting of an oxidation of graphite powder followed by an exfoliation then a reduction back to graphene, seems to be the best access for the production of large amount of single layers or at least graphene nanoplatelets formed by several graphene sheets stacked together. But this process, intented to provide large amounts of monolayers, generates, as mentioned by Singh et al. [85], structural defects that disrupt the electronic structure of graphene. Raman spectroscopy that has proved to be one of the most powerful technique for the characterization of carbon-based materials [86], clearly indicates the presence of imperfections [87,88,89]. The Raman spectrum of graphite excited at 633 nm, displays two main bands located at 1581 cm^−1^ and 2687 cm^−1^ called the G and G′ (or 2D) bands respectively. The first one, present in all carbon materials, corresponds to the degenerate in-plane E_2g_ optical mode at the center of the Brillouin zone while the second one, assigned to a second-order Raman scattering, corresponds to the first overtone of the disorder-induced D band, and is observable even in the absence of defects (Figure 12a). Graphene oxide (GO) exhibits, besides the G band, broadened and shifted to 1590 cm^−1^, a strong band at 1337 cm^−1^ (D band) associated with the presence of disorder and of amorphous carbon (Figure 12b). Moreover, the G′ band that has been shown to give information on the number of layers and on stacking order in graphene systems [90], is markedly broadened. As seen in Figure 12c, the Raman spectrum of GO is similar to that of highly defective carbons like CB. Stankovich et al. [87] have shown that the reduction of the electrically insulating graphene oxide, intended to restore the electrical properties of the graphitic network, leads to an increase of the I_D_/I_G_ intensity ratio compared to that of GO, suggesting to the authors a further decrease in the average size of the sp^2^ domains upon reduction of GO. 

**Figure 13:**
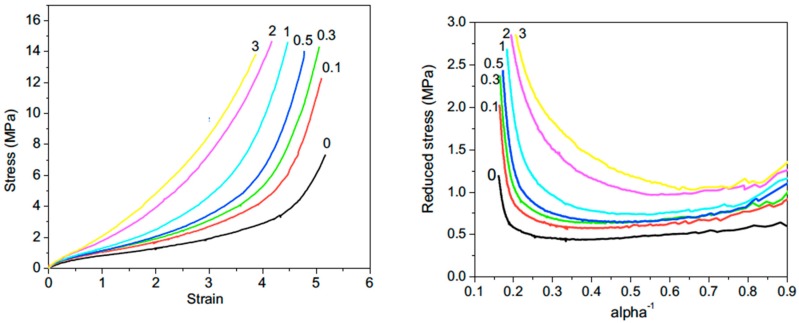
Stress-strain curves (left) and Mooney-Rivlin plots (right) of unfilled NR and NR filled with surface functionalized graphene oxide with bis(triethoxysilylpropyl)tetrasulfide. The numbers in the figure indicate the weight fractions of filler in the nanocomposites. Reproduced with permission from [91]. Copyright Elsevier, 2013.

Regarding NR, an interesting approach is that of Wu et al. [91] who graft onto the surface of GO, a silane coupling agent (bis(triethoxysilylpropyl)tetrasulfide (BTESPT)), generally used in the case of poor adhesive qualities of a filler for a polymer. This coupling agent, is able to establish molecular bridges at the interface between the polymer matrix and the filler surface as in the case of a silica-filled hydrocarbon rubber. By this way, the hydrophilic character of GO, not compatible with the hydrophobic NR, is reduced, allowing a better dispersion of the filler in the host polymer. It is shown that the mechanical properties of NR are significantly improved at very low filler loading since a 100% inrease in the tensile strength and a 66% improvement in the tensile modulus are achieved with only 0.3 wt% of grafted GO. And as already observed in NR/clay and NR/CNT systems, the onset of crystallization is observed at a lower strain value than that of the unfilled matrix (Figure 13). These improvements, much more important than those obtained with CNTs [69], may be understood by an increase in the cross-linking density caused by the reaction of the polysulfide groups of the coupling agent with the rubber molecules. On the other hand, the high values of the tensile strength with regard to those reported for NR/CNT composites reflect a better dispersion of the graphene derivative. The same group [92] carried out further investigations including dielectric relaxation and synchroton WAXS experiments as well as Raman mapping and Raman measurements under strain intended to bring new evidence for strong polymer-filler interaction. 

Other attempts using GO as a reinforcing filler for NR are reported in the literature [93,94]. Wu et al. [93] have analyzed NR/GO nanocomposites with three different size GO prepared through the latex mixing method. They demonstrate that the smallest size of GO sheets yields the higher enhancement in the modulus, the higher Payne effect and induces the earlier crystallization of NR. In the work of Zhang et al. [94], the GO/NR samples are prepared by using a latex in which the cross-linking agent was already included. It is shown that the initial slopes of the stress-strain curves and the storage modulus in the rubbery region, increase sharply with the filler content. High electrical conductivity is achieved by carrying out the reduction of GO after the formation of the composites by immersing the film in HI acid. By this way, the flocculation and re-stacking of the reduced platelets are expected to be avoided. A modified latex mixing method was also used to prepare graphene/natural rubber (GE/NR) composites [95]. In the method, a mixture of an aqueous GO dispersion and a NR latex was co-coagulated with saturated solution of sodium chloride then hydrazine hydrate was used to reduce to GO in situ. Incorporation of GE into NR leads to strong increase in modulus and a loading as low as 0.5 phr imparts to the elastomeric matrix a 80% increase in the initial tensile modulus [96]. Application of the Halpin-Tsai model [97] to the storage modulus at 10 °C of the GE/NR composites allows the authors to deduce a shape factor of 719 thus suggesting a molecular level dispersion of GE in the elastomeric matrix. Most recently, La et al. [98] have used a one-step approach for the mass production of graphene nanoplatelets (GNPs) by direct chemical exfoliation of natural graphite. The elongation at break and tensile strength are seen to increase upon addition of 0.3 phr of GNPS then decrease at higher loadings. Surprinsingly, the authors do not report any increase in stress at a given strain which is usually one of the most striking change brought about by the presence of reinforcing particles in elastomeric materials.

The above results show that most studies demonstrate the high potential of graphene as a nanofiller for rubber materials. Improved properties of the nanocomposites were obtained at very low graphene loadings but despite the different approaches aiming at obtaining nanocomposites with homogeneous dispersions of nanolayers, it seems that layers stacking and agglomeration prevents the full realization of the graphene capability. 

## 5. Conclusions

This paper reviews some studies carried out on natural rubber compounded with fillers expected to provide nanoscale reinforcement. They include particles generated in situ via the sol-gel process, clays and carbon nanomaterials. Improved mechanical properties are imparted by all types of fillers to the rubbery matrix at very low filler loading compared to the much larger amounts of conventional fillers used to obtain the desired properties. The reduction in the filler content of nanofillers leads to lighter weight and better processability of the materials which is of major interest in industrial applications. Almost all studies show that the crystallization of NR starts at a lower deformation in the presence of filler as a result of overstraining of rubber chains by strain amplification effects and also of a higher chain orientation in the direction of strain.

Conclusions drawn from the reported studies can be summarized as follows:

The greatest advantage of the sol-gel method, considered as an alternative technique for in situ silica loading of elastomers, is to provide a more homogeneous dispersion of the filler in the matrix than that obtained with conventional mixing techniques thus ensuring a better performance of the nanocomposites. This in situ technique, can be carried out under mild synthetic conditions, before and after the cross-linking process. Conducted in vulcanized NR films, it provides almost ideal dispersions because the filler structures grow within the preformed organic network causing a steric limitation for cluster growth. The generated spherical silica particles impart to the elastomeric matrix a substantial reinforcement despite the poor affinity of silica for NR. Interfacial dynamics and especially confinement of polymer chains on the filler surface and strain amplification effects have to be taken into account to explain the main features caused by the in situ filling process. In the unvulvanized NR, silane coupling agents are often used to prevent agglomeration of the nanoparticles due to the incompatibility between the hydrophilic inorganic fillers and the hydrophobic polymer matrix. The main obstacle for the application of the sol-gel processes at a larger scale is the use of expensive alkoxide precursors in the liquid state. 

Another parameter that plays a crucial role in the performance of the rubber material is the aspect ratio of the nanofillers. It is the case of layered fillers such as clays or graphitic structures and of rod-shaped particles such as clay fibers or carbon nanotubes. These anisoptropic particles are able to orientate along the direction of stretch and can form a filler network at low filler content which strongly affects the mechanical behavior of the composite and provide electrical conduction in the case of carbon nanomaterials. 

Owing to their large availability, low cost, high surface area and possible full separation (exfoliation) of silicate layers, layered clays have attracted a huge interest as reinforcing agents for rubber materials. But despite the fact that a complete exfoliated structure is rarely obtained, most studies report significant enhancement in the mechanical properties and outstanding barrier properties mainly explained by the increased aspect ratio of the layered structure that reduces the diffusion of molecules causing a more pronounced tortuous path. 

Among clays, needle-shaped fibers of sepiolite, organically modified for a better compatibility with the hydrophobic matrix, have been seen to impart to NR a stronger reinforcement than the spherical particles. It is due to the high anisotropic character of the rod-like particles and to their ability to align in the direction of stretch. 

Besides their intrinsic exceptional stiffness and strength, one major advantage of carbon nanomaterials over "white" fillers is to provide electrical conductivity when incorporated into elastomers usually considered as electrical insulators. The electrical percolation threshold associated with the formation of a filler network is achieved at tiny concentrations of nanofillers compared to conventional carbon black particles. 

Considerable improvement in stiffness is observed upon addition of carbon nanotubes to the NR matrix but also a strong reduction in the strain at rupture with the nanotube loading ascribed to the presence of bundles. In fact, until today, one of the main challenges is to overcome agglomeration of nanotubes due to van der Waals interactions between individual tubes. 

One way to improve the nanotube dispersion is to incorporate to the matrix a second filler on account of possible synergistic effects between two different filler morphologies. The dual filling approach aims to combine the benefits of each reinforcing agent in order to further enhance the matrix properties. An interesting application of the use of hybrid filler is the incorporation of a small amount of carbon nanotubes into silica-filled systems to bring electrical conductivity to the insulating silica-filled rubber.

Due to its extraordinary characteristics such as stiffness, strength and electrical conductivity, graphene, the monolayer of graphite, is expected, if complete exfoliation of graphite is achieved, to yield high-performance rubber nanocomposites with much better properties than that imparted by the other nanofillers. The most common approach for the production of graphene sheets is the exfoliation and reduction of graphite oxide. But this chemical process often generates single sheets in addition to graphene platelets and like with other fillers, the extent of property improvement of the resulting composite depends on the state of dispersion. So the performance of this outstanding filler is still limited by inhomogeneous dispersions. Challenges in processing and dispersion are strongly required for the full realization of the graphene potential.
